# Case of Twins

**Published:** 1844-10-19

**Authors:** Chas. D. Meigs

**Affiliations:** Philadelphia


### THE MEDICAL EXAMINER,

### And Record of Medical Science.

Vol. VII.] PHILADELPHIA, SATURDAY, OCTOBER 19, 1844. [No. 21.

### CASE OF TWINS,

*With locking of the fore-arm of one of them, and fracture of the humerus—successfully treated.*

BY PROFESSOR MEIGS.

*Philadelphia, Sept.* 29th, 1844.

To the Editor of the Medical Examiner:

SIR—On the 13th inst., I attended Mrs. H—— South 11th street, in labour with twins. Both of the children came footling, and were of good size. The eldest, which was safely delivered, was followed in a few minutes by the second child, which came very well, until I found that the right shoulder would not descend in consequence of the fore-arm having been keyed behind the infant's neck. I made various attempts to get the arm unlocked, but it was so jammed against the side of the pelvis, that I failed to get it down, though I used Dr. Dewee's method of pushing the shoulder in the direction of the child's sternum. Finding that the pulsations of the cord began to give signs that the child would be lost, and that the spasmodic twitching of its legs also indicated its extreme peril, I said to the nurse that I feared the bone would break before I could deliver. I at length got the limb down, by pressing with my finger near the elbow—but the os humeri was fractured.

Upon carefully examining the child soon after its birth, I could not clearly detect the signs of the fracture, the arm being much swelled and contused. The next day, however, I found it perfectly flexible at the fractured point.

It was on the 14th inst. that I put the limb in a carved splint, and I find this morning that union is complete, which is sixteen days that the bone has been under treatment.

The motive for offering you this article is chiefly that you may put on record the fact that in the newborn child the progress of ossification, in a fracture of the os humeri, is so speedily effected. Indeed, I am of opinion that the arm was firm on Thursday last, which was 13 days only after the first dressing.

Permit me to suggest that an accoucheur who feels that his manœuvre is about to endanger the limb of a fœtus in utero, whose life he desires to save, or whose mother must be saved, *coute qui coute*, ought, in justice to himself as well as his art, to announce that the risk and the necessity of running it, are equally great, so that the parents or friends may not have any excuse, or indeed inclination, to charge him with carelessness or improper violence.

I am sir, your obed't serv't,

CHAS. D. MEIGS.

